# A Perspective on the Role of Microbiome for Colorectal Cancer Treatment

**DOI:** 10.3390/cancers13184623

**Published:** 2021-09-15

**Authors:** Sanjna Kalasabail, Jared Engelman, Linda Yun Zhang, Emad El-Omar, Howard Chi Ho Yim

**Affiliations:** Microbiome Research Centre, St George and Sutherland Clinical School, The University of New South Wales, Sydney 2052, Australia; s.kalasabail@student.unsw.edu.au (S.K.); jared.engelman@health.nsw.gov.au (J.E.); lzhan170@jhmi.edu (L.Y.Z.); e.el-omar@unsw.edu.au (E.E.-O.)

**Keywords:** colorectal cancer, microbiome, synbiotics, 5-fluorouracil, capecitabine, oxaliplatin, leucovorin, irinotecan, chemotherapy, toxicity

## Abstract

**Simple Summary:**

Colorectal cancer is the third most diagnosed cancer worldwide and contributes significantly to global mortality and morbidity. The gut microbiome, composed of the trillions of microbes endemic to the human gastrointestinal tract, has been shown to be implicated in colorectal cancer oncogenesis; however, the roles of microbiota and dysbiosis in CRC treatment remain poorly understood. This review sought to characterize this relationship and in doing so, identify how these interactions may inform future treatments in the form of synbiotics designed to alter the host microbiota to achieve optimized treatment outcomes.

**Abstract:**

In healthy hosts, trillions of microbes colonise the gut and oral cavity in a well-balanced state, maintaining a mutually beneficial relationship. Loss of this balance, termed dysbiosis, is strongly implicated in the pathogenesis of colorectal cancer (CRC). However, the roles of microbiota and dysbiosis in CRC treatment remain poorly understood. Recent studies suggest that the gut microbiota has the ability to affect the host response to chemotherapeutic agents by enhancing drug efficacy, promoting chemoresistance and mediating chemotherapy-induced toxicity and side effects via a variety of mechanisms. Several other studies have also proposed manipulation of the microbiota to optimise CRC treatment. In this review, we summarise the current advancement of knowledge on how microbiota and CRC treatments interact with each other and how this interaction may shed some light on the development of personalised microbiota manipulations that improve CRC treatment outcomes.

## 1. Introduction

### 1.1. Epidemiology

Colorectal cancer (CRC) is a major public health problem, being the third most commonly diagnosed malignancy and the second most common cause of cancer-related death worldwide in 2020 [1,2,3]. CRC is an aetiologically heterogenous disease that arises through three major pathways–the adenoma-carcinoma sequence, the serrated and the inflammatory pathways and is also associated with certain genetic factors [4]. However, most CRC cases are sporadic in nature, emerging from a complex interplay between modifiable environmental risk factors attributable to westernisation [4,5]. As such, the global burden is expected to further increase as a result of the growth and ageing of the population and the adoption of westernized behaviours and lifestyles [6]. A topical area in CRC research has emerged from recent studies demonstrating a state of imbalance or dysbiosis in CRC patients, implicating the gut microbiota in colorectal carcinogenesis [7,8]. There is wide geographical variation with over two-thirds of its incidence and approximately 60% of total deaths occurring in countries with a high or very high human development index [1]. The lifetime risk of developing CRC in many regions is roughly 5%, with deaths occurring in just under half (45%) of those diagnosed despite treatment [9]. There is a poor prognosis for patients with metastatic CRC with a median 5-year survival rate of 12.5% in the USA, underlining the necessity of developing more effective treatments [10].

### 1.2. The Microbiome

The human body houses a vast and highly integrated microbial community of bacteria, fungi, viruses, archaea and parasites, collectively termed the microbiota. The bacteria are predominately represented by the phyla *Firmicutes, Bacteroidetes* and *Actinobacteria* [11]. The gastrointestinal tract (GIT) contains over 100 trillion microorganisms and is the site of principal communication between host cells, the immune system and the microbiota [11,12,13]. The gut microbiota has co-evolved with the host and plays an active role in regulating metabolism and immunity, and maintaining homeostasis and nutritional absorption [14,15]. The proposed causative role of the microbiota in carcinogenesis is through dysbiosis-related inflammation and biosynthesis of carcinogens, with shifts in the microbiome commencing as early as in precancerous adenomas [15,16,17]. Next-generation sequencing technologies have been used to study the microbiome composition [18]. Analysis of the microbiome entails sample collection and processing, next-generation sequencing and a bioinformatics analysis [19]. Many bodily samples can be collected and studied, such as saliva, faeces, tissue biopsies, intestinal fluids, buccal swabs, skin and the vaginal swab [19]. Nucleic acids of these samples can be sequenced by gene amplicon sequencing and whole-genome shotgun metagenomics sequencing [20]. Gene amplicon sequencing is used to identify microbial composition by sequencing the hypervariable regions of a target gene that are conserved among taxa in a particular kingdom of microorganisms. For example, the hypervariable region of 16S rRNA can be amplified and sequenced to determine the bacterial composition in the given samples. [20] This technique is mostly employed on DNA samples that have a high host to microbial DNA ratio. These samples include tissue biopsies and skin. Conversely, unlike 16S rRNA sequencing, metagenomics uses genome-wide shotgun sequencing, targeting the whole genome to provide a superior taxonomic resolution [20]. This technique is mostly employed on DNA samples that have a low host to microbial DNA ratio. These samples include faeces, intestinal fluid, buccal and vaginal swabs.

### 1.3. Treatment for CRC

Despite being the most effective, surgical interventions are unable to completely cure CRC, and thus post-operative adjuvant chemotherapy has emerged as an essential therapeutic option to prevent recurrence and metastasis [21]. CRC is commonly treated with cytotoxic agents that interfere with DNA replication such as 5-fluorouracil (5-FU), capecitabine and oxaliplatin, as well as regimens such as FOLFOX (5-FU and oxaliplatin), FOLFIRI (5-FU and leucovorin and irinotecan) and XELOX (oxaliplatin and capecitabine) [21,22]. Most forms of chemotherapy target tissues that have a high rate of cell turnover and division, thereby affecting other normally dividing cells with a similar division rate. This may cause severe side effects such as gastrointestinal mucositis requiring dose reduction or deferral of treatment, resulting in poorer prognosis [23].

Immunotherapy is another mode of systemic cancer therapy that drives a “tumour-specific” immunity by establishing a durable population of highly active T cells that can target and lyse tumour cells [24,25]. The efficacy of current immunotherapies vastly depends on the tumour category of the cancer. CRC can be classed as being mismatch-repair-deficient or high microsatellite instability (dmmR-mSI-H) or more commonly, as mismatch-repair-proficient or microsatellite instability-low (pmmR-mSI-H) [10]. Current immunotherapy utilises monoclonal antibodies targeting programmed cell death 1 (PD1) such as pembrolizumab and nivolumab, which have demonstrated improved survival only in metastatic dmmR-mSI-H CRC [10]. However, targeted immunotherapies functioning beyond PD1 blockade with greater tumour efficacy are ongoing.

Recent studies have investigated the relationships between the microbiome and the outcome of cancer therapy such as efficacy and toxicity. Understanding the relationship between the microbiome changes before and after CRC treatment will hold the key to reducing toxicity and improving efficacy of the treatments. This review will attempt to address these aspects.

## 2. Effect of Microbiome on Chemotherapy Efficacy and Toxicity

### 2.1. Effects of Microbiome on Chemotherapy Efficacy

**Figure 1:**
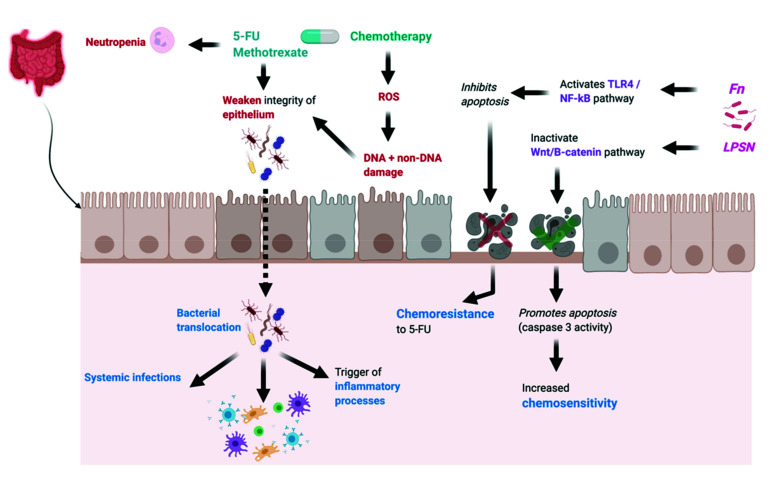
The effect of microbiome on chemotherapy efficacy and toxicity. The gut microbiota can affect the host response to chemotherapeutic agents by facilitating drug efficacy, promoting chemoresistance and/or mediating chemotherapeutic toxicity and side effects. On the other hand, the efficacy of anticancer therapy is dependent on microbiota-mediated innate and adaptive immune responses. As a whole, chemotherapy induces ROS-mediated DNA and non-DNA damage, resulting in bacterial translocation across the intestinal epithelium. This in turn induces an inflammatory response and can provoke systemic infections. Abbreviations: 5-FU, 5-fluorouracil; DNA, deoxyribonucleic acid; *Fn*, *fusobacterium nucleatum*; *LPSN*, *lactobacillus plantarum supernatant*; ROS, reactive oxygen species. The bolded arrows represent the effects of the microbiome and chemotherapy, and the broken arrow represents bacterial translocation.

The gut microbiota has the ability to affect the host response to chemotherapeutic agents by facilitating drug efficacy, promoting chemoresistance and/or mediating chemotherapeutic toxicity and side effects [26,27]. The translocation, immunomodulation, metabolism, enzymatic degradation and reduced diversity and ecological variation (TIMER) mechanistic framework (Figure 1) has been proposed as a model to explain the variety of mechanisms that allow the gut microbiota to modulate chemotherapy agents [27]. These mechanisms are important in understanding chemotherapy success and failure in a variety of tumours and specifically in colorectal cancer (CRC).

Two elegant studies using high-throughput sequencing in a C. elegans model [28,29] highlighted the importance of host–microbe interactions in promoting the anti-tumour efficacy of fluoropyrimidine type chemotherapy agents—those that are standard as a first line treatment against CRC. Fluoropyrimidines such as 5-fluorouracil (5-FU) are thought to exert their anticancer effects by inhibiting thymidylate synthase, thus impeding nucleotide biosynthesis and hence cell division [28]. Interestingly, a study by García-González et al. [29] suggested that 5-FU and 5-fluoro-2′-deoxyuridine (FUDR) act by affecting ribonucleotide rather than DNA metabolism, a process that is dependent on active bacterial metabolism. They showed, within their model, that *Escherichia Coli (E. coli)* use an inherent pathway to convert 5-FU and FUDR into fluorouridine monophosphate (FUMP), an analogue of uridine monophosphate (UMP) that has been shown to block de novo pyrimidine synthesis. Scott et al. [28] echoed this in their C. elegans model, showing that inhibition of bacterial ribonucleotide metabolism significantly antagonised drug efficacy. Additionally, their results imply that the diversity of the nematode’s microbiome is critical to the host response to fluoropyrimidines, with pharmacodynamics of the drug varying as much as 40-fold with different bacterial strains and up to 256-fold with disruption of bacterial metabolism. This symbiosis between the host microbiome and fluoropyrimidine efficacy is further supported in a mouse model reported by Yuan et al. [30]. In their study, they compared the anti-CRC efficacy of 5-FU in mice treated with a cocktail of antibiotics of vancomycin, ampicillin, neomycin and metronidazole with those without these treatments. Their results showed that after 35 days, the tumour volume was significantly lower in the mice that were not treated with antibiotics compared to those that were. They concluded that antibiotic administration caused the disruption of the gut microbiota and thus reduced 5-FU efficacy, implying that microbiome dysbiosis is unfavourable to chemotherapeutic outcomes. This was echoed in Wang et al.’s mouse model, which demonstrated that microbiota dysbiosis induced by dysfunctional TGF-ß signalling was more likely to develop CRC resistant to 5-FU [31]. Other bacteria endemic in the microbiome such as *Lactobacillus plantarum supernatant* (LPSN) may play a role in increasing chemosensitivity in 5-FU CRC resistant cells (Figure 1). This mechanism by which LPSN is able to improve chemosensitivity is multifaceted and may involve inhibiting expression of particular biomarkers on cancer stem cells, selectively inactivating the Wnt/B-catenin signalling pathway and promoting cell death and apoptosis by inducing caspase 3 activity [32,33].

The efficacy of anticancer therapy is proposed to depend on microbiota-mediated innate and adaptive immune responses [34,35]. In a study by Lehouritis et al. [36], 30 drugs were tested in vitro in the presence of non-pathogenic *E. coli* or *Listeria welshimeri*. While 10 of the drugs were found to be inhibited by one or both species, the efficacy of six of the drugs was enhanced. In another experimental study, the anti-tumour effect of cyclophosphamide was attenuated when the microbiota was altered [35]. Sterilisation by broad spectrum antibiotics or vancomycin to specifically targeted gram-positive bacteria resulted in reduction of cyclophosphamide’s ability to induce an inflammatory response. Cyclophosphamide induces CD4+ T cells in the spleen to become IL-17 producers, and the gut microbiota is vital for this process. Antibiotic-treated and germfree mice experienced reduced anti-tumour effects in response to cyclophosphamide treatment [35]. Similarly, Iida et al. [34] treated mice with an antibiotic cocktail of vancomycin, imipenem, and neomycin in drinking water for 3 weeks before inoculating with MC38 colon tumour cells. The mice subsequently underwent immunotherapy and chemotherapy with either oxaliplatin or cisplatin. Mice treated with antibiotics or germfree mice had an attenuated response to therapy. In these mice, tumour-infiltrating myeloid-derived cells produced reduced levels of several cytokines (including TNFα) as well as reactive oxygen species (ROS) after treatment. Hence, an intact commensal microbiota may modulate myeloid-derived cell functions in the tumour microenvironment and is crucial for the early cytotoxic response to cancer immunotherapy and chemotherapy [34]. Two commensals in particular, *E. hirae* and *B. intestinihominis*, have been shown to stimulate specific-memory Th1 cell immune responses, leading to a longer progression-free survival in advanced lung and ovarian cancer patients treated with cyclophosphamide [37]. Moreover, it has been shown that colon cancer prognosis is in a large way dictated by the abundance and functional response of tumour infiltrating lymphocytes (TIL), including follicular T helper cells, in addition to the efficacy of chemotherapy-induced immune responses [38,39,40]. Roberti et al. demonstrated the importance of the ileal microbiota in dictating tolerogenic versus immunogenic ileal intestinal epithelial cell death and the accumulation of follicular T helper cells in both patients and mice with colorectal cancer [41]. They found that the immunogenicity of oxaliplatin therapy is enhanced when ileal intestinal epithelial cells are colonised with immunogenic commensals, particularly *Bacteroides fragilis (B. Fragilis)* and *Erysipelotrichaceae*. In the presence of these bacteria, chemotherapy-induced apoptotic ileal crypt cells produced interleukin-1R1 and interleukin-12, which in turn elicited a PD-1+ follicular T helper cell response, thus improving chemotherapeutic efficacy. Their findings not only show the importance of immunogenic ileal apoptosis in the prognosis of chemotherapy-treated CRC, but they also outline the vital role the microbiota play in dictating this response, further emphasising the influence of the microbiota in modifying the efficacy of anticancer therapy [41].

Contrasting the perceived symbiosis between an intact microbiome and chemotherapy efficacy, a number of studies have suggested that the host microbiota might serve to increase the chemoresistance of 5-FU. While *Fusobacterium Nucleatum* (*Fn*) has been implicated in the pathogenesis of CRC, Zhang et al. [42] show that *Fn* might play a role in CRC chemoresistance to 5-FU through activation of the TLR4/NF-kB pathway causing upregulation of BIRC3, an inhibitor of apoptosis (IAP) protein on CRC cells. They showed that *Fn* abundance was correlated with chemoresistance and identified high amounts of *Fn* as an independent risk factor for recurrence in advanced CRC patients. Yu et al. [43] also demonstrated a mechanistic role for *Fn* in promoting resistance to 5-FU and oxaliplatin chemotherapy regimens. They showed that *Fn* activates TLR4 and MYD88 immune signalling to inhibit the expression of microRNA (miRNA)-18a and miRNA-4802. This in turn suppresses the autophagy pathway and the apoptosis of the colon cancer cells induced by 5-FU and oxaliplatin, leading to chemoresistance (Figure 1).

Irinotecan (CPT-11) is a commonly used chemotherapeutic agent that often results in gastrointestinal side effects as a result of its unique metabolism. Bacterial β-glucuronidases in the colon are crucial to the conversion of CPT-11 to its active agent SN-38, which is responsible for anti-tumour activity as well as dose-limiting toxicity [32]. SN-38 undergoes hepatic glucuronidation and is secreted into bile as the inactive glucuronide SN-38G [44]. Bacterial β-glucuronidases in the colon deconjugate SN-38G, exposing the intestinal epithelia to SN-38, thus causing gut toxicity as well as allowing bacterial organisms to translocate, causing systemic infection and sepsis [45,46,47]. Antibiotic prophylaxis not only reduces SN-38 concentration, but also diarrhoea in animal and human models [47,48].

Increasingly, immunotherapy is becoming a major treatment modality for a subset of CRC subtypes. In particular, immune checkpoint inhibitors (ICIs) such as anti-PD1 and anti-CTLA-4 treatments have shown some efficacy in dMMR-MSI-H CRC [10]. The microbiome may be able to modulate the host response to immunotherapy to increase its antineoplastic activity. Specific bacteria within the microbiota have been shown to correlate positively with immunotherapeutic response, and mouse models have demonstrated enhanced immunotherapy efficacy with particular bacterial microbiota strains [34,49,50]. Currently, these findings have not been replicated in human studies, and therefore the importance of these bacterial strains in optimising clinical outcomes is still unknown.

The studies above demonstrate the diverse impact gut microbiota can have across a spectrum of antineoplastic treatment in CRC. The studies above have highlighted potential avenues to harness the microbiome by creating a microenvironment that is beneficial to increasing treatment efficacy; however, the challenge lies in replicating these results in human trials and documenting improved outcomes for these patients. Understanding the role of the gut microbiota in influencing the host response to anti-tumour treatments, including chemotherapy and new immunotherapeutic approaches, will be essential in developing personalised treatments to achieve optimal efficacy and tumour clearance. Using this knowledge as a basis to investigate the synergistic role of faecal microbiota transplantation or symbiotics with anticancer therapy is an exciting next step in combatting CRC resistance and mortality.

### 2.2. Effects of Microbiome on Chemotherapy Toxicity

The cytotoxic effects of chemotherapy give rise to a wide range of toxicities, including the inflammatory condition of mucositis. Current drugs or other approaches to counteract chemotherapy-induced adverse effects are often incompletely effective, frequently do not address potential longer-term sequelae or may even induce other side-effects that only add to patient discomfort [51]. Chemotherapy drugs such as 5-FU and methotrexate are highly toxic for intestinal epithelial cells lining the gut mucosa and other cells with high proliferation rates [52]. These drugs cause neutropenia and weaken the integrity of the epithelial barrier, facilitating bacterial translocation across the gut mucosa, allowing for systemic infections and triggering of inflammatory processes [52,53]. Such processes are associated with mucositis, which affects up to 80% of patients depending on treatment regimen [54]. These toxicities increase the risk of infections and interfere with the provision of optimal cancer treatment [55].

Chemotherapy-induced gastrointestinal toxicity (CIGT) covers a constellation of cancer treatment-related adverse events [54]. CIGT is characterised by painful, ulcerative lesions throughout the gastrointestinal tract that specifically affect the non-keratinized mucosa [55]. CIGT is associated with an increased risk of other adverse events such as infection and diarrhoea [54]. Though it is a self-limiting condition, debilitating symptoms including clinically significant gastrointestinal bleeding and pain as well as complications requiring increased hospital stays and parenteral nutrition translate to reductions in antineoplastic therapies and hence reduced survival [54,56].

Mucositis is one of the most common and significant toxicities of chemotherapy, with an incidence of 15% among those receiving low-risk treatments and between 60–100% of those treated with high-dose CT, radiotherapy and bone marrow transplantation [57]. However, its pathophysiology is multifaceted and thought to be associated with dysbiosis in the host. Mucosal ulcerations are suggested to develop in five stages [23,58]. The first stage begins within seconds of exposure to chemotherapy or radiation and is characterised by the generation of ROS, direct DNA and non-DNA damage and activation of the immune response [57]. This activates the inflammasome and pyroptosis, followed by proinflammatory transcription factors such as nuclear factor- kB (NF-kB) [59,60]. NF-kB-mediated gene expression causes the release of pro-inflammatory cytokines, including tumour necrosis factor-α (TNF-α), interleukin (IL)-1β, and IL-6, which further amplify damage to mucosal cells and breakdown of connective tissue [57,59,60]. These stages are asymptomatic as they occur in a continuous loop, amplifying the effects and causing progressive tissue injury, and thus mucositis becomes clinically evident during the development of ulceration and inflammation [59]. It is in these stages that the integrity of epithelial tight junctions is suggested to be compromised, resulting in increased intestinal permeability, allowing for translocation of bacteria and thus altered gut microbial composition and an increased risk of bacteraemia and sepsis (Figure 1) [59,61].

Studies have identified that microorganisms occupy niches that are essential for the development of mucositis [52]. It is thought that the intestinal microbiota exerts a dual role in the development of mucositis either through the production of beneficial metabolites such as butyrate to reduce inflammation or via the deconjugation of SN-38 glucuronide by β-glucuronidase-producing bacteria such as Enterobacteria that propagate intestinal damage [62]. Specific shifts in microbial composition have been observed in the following clinical and pre-clinical studies.

Germ-free (GF) pre-clinical studies have hypothesised that the microbiome is heavily implicated in mucositis development, with GF mice being more resistant to 5-FU-induced mucositis [62,63]. Stringer and Al-Dasooqi [64] reported a marked change in the intestinal microbiome in patients with chemotherapy-induced gastrointestinal mucositis, particularly a decrease in beneficial commensals such as *Bifidobacterium* spp., *Lactobacillus* spp., *Bacteroides* spp. and *Enterococcus* spp. This disease state was also associated with a shift towards pathobionts such as *E. Coli* and *Staphylococcus* spp. The finding of *Enterobacteriaceae* enrichment following chemotherapy mimics the findings of a preclinical IBD study, suggesting inflammation occurs in these patients [65]. Similarly, a longitudinal study of subjects undergoing 5-FU or doxorubicin-based chemotherapy by Hong et al. [55] found an association between chemotherapy-induced oral mucositis and dysbiosis. The shifts to mucositis-associated bacteriome, as measured in saliva and buccal mucosa samples, entailed a depletion of symbiotic bacteria from the genera *Streptococcus*, *Actinomyces*, *Gemella, Granulicatella* and *Veillonella* as well as an enrichment of Gram-negative bacteria such as *Fusobacterium nucleatum* and *Prevotella oris*. These shifts were attributed to the inflammation-associated oral dysbiosis rather than the direct antibacterial effects of 5-FU via an unknown mechanism, highlighting a potential avenue for future research.

Chemotherapy may also induce changes that confer long term toxicity to the patient that extend beyond local mucositis. Chemotherapeutic DNA damage within the intestinal microbiota is likely to activate the bacterial SOS response system, a global response to DNA damage in which the cell cycle is arrested and DNA repair occurs via a multi-step process. The SOS response system has been shown to increase bacterial mutagenesis via low-fidelity DNA polymerase repair, resulting in de novo antimicrobial resistance [61]. Mechanistically, Thi et al. [66] showed that eight different antibiotics induced mutagenesis in *E. coli* with trimethoprim, individually or in combination with sulfamethoxazole, producing the highest level of mutagenicity. They postulated that the reduction in the thymidine nucleotide pool may work synergistically with SOS response activation to increase bacterial mutagenesis. Chemotherapeutic agents such as 5-FU and capecitabin, both commonly used in CRC treatment, block thymidylate synthase, thus altering thymidine nucleotide levels in a similar fashion to trimethoprim/sulfamethoxazole, thus increasing the possibility of bacterial mutagenic change resulting in de novo antimicrobial resistance.

It is likely that any chemotherapeutic agent that causes DNA damage may activate the bacterial SOS response system and increase bacterial mutagenicity. However, it is still unknown which agents potentiate the greatest mutagenic effect resulting in clinically significant antimicrobial resistance [61]. The studies above suggest that agents blocking thymidylate synthase may confer the greatest risk of creating de novo antimicrobial resistance, and thus future research should initially focus on chemotherapeutic agents that have this property, such as 5-FU and capecitabin.

### 2.3. Effects of Microbiome on Immunotherapy Efficacy

The gut microbiota has been heavily implicated in the functioning of the immune system, as demonstrated by a multitude of GF pre-clinical studies. GF mice have been demonstrated to have poor immune functioning secondary to factors such as fewer and smaller goblet cells and Peyer’s patches, lack of lymphoid follicles in the lamina propria, reduced CD4^+^ T cells, plasma cells, and decreased IgA production, which can be reversed following colonisation with commensal bacteria [67]. As a result, studies have explored the role of the microbiota and its influence on immunotherapy efficacy.

Vetizou et al. [68] showed that the efficacy of the monoclonal antibody ipilimumab against anti-CTLA-4 relied on the presence of *Bacteroides* species—specifically *B. thetaiotaomicron* or *B. fragilis* in patients with melanoma. A novel study utilising 18 “bulk” RNA-seq datasets (*n* = 2269) and four single-cell RNA-seq datasets to generate a “Signature associated with FOLFIRI resistant and Microenvironment” (SFM) illustrated that SFM subtypes were associated with differing outcomes, including gut microbiome composition, and this impacted treatment response in colorectal cancer [69]. Specifically, SFM-C (a subtype of SFM based on SFM signature discriminating between the tumour microenvironment and drug sensitivity) increased abundance of *Bacteroides thetaiotaomicron*, *Fn*, and *B. fragilis,* and was shown to be responsive to immunotherapy. Conversely, SMF-F was enriched with *Corynebacterium aurimucosum* and *Pseudomonas putida* and was not responsive to immunotherapy [69]. It was hypothesised that the tumour microenvironment (TME) of SFM-C was enriched with the MSI phenotype and so the immune suppression could be blocked by immune inhibitors, whilst the TME of SFM-F was enriched with a phenotype that could also cause immunosuppression but could not be reversed by immune inhibitors. These findings are significant as they demonstrate an avenue for microbiota modification to improve treatment response; however, further research on how this directly applies to CRC is warranted given the relative novelty of immunotherapy in this patient cohort.

### 2.4. Effects of Microbiome on Immunotherapy Toxicity

Reported adverse outcomes following immunotherapy treatment include a lack of response, immune-related adverse events such as diarrhoea or colitis and acceleration of tumour progression [70,71]. It is thought that these unfavourable outcomes are due to dysbiosis.

A study analysing faecal samples of 26 patients at baseline and prior to ipilimumab treatment demonstrated that a baseline microbiota with enriched *Faecalibacterium* genus and other *Firmicutes* had better response, demonstrated by a longer progression-free survival than those with a baseline enrichment of *Bacteroides* in patients with melanoma [72]. Importantly, such generalisations may not hold at lower levels of taxonomy, as Streptococcus (taxa within Firmicutes) is actually associated with poor antitumour effect [73]. Studies have demonstrated that enrichment of *Bacteroides* is associated with less frequent occurrence of ICI toxicity such as colitis when treating melanoma [72,74,75]. A recent review identified several studies that highlighted the correlation, in patients with melanoma, between the *Ruminococcaceae* family of the Firmicutes phylum with therapeutic efficiency and treatment-linking toxicity of ICI [73].

Although there is a paucity of studies exploring the link between immunotherapy in CRC and the microbiome, current research has demonstrated a relationship between the microbiome and immunotherapy toxicity and efficacy in other cancers, in particular melanoma. This paucity highlights the need for a more systematic approach in analysis of specific disease processes studied and the sample types used.

## 3. Effects of Antineoplastic Treatment on the Microbiome

**Figure 2:**
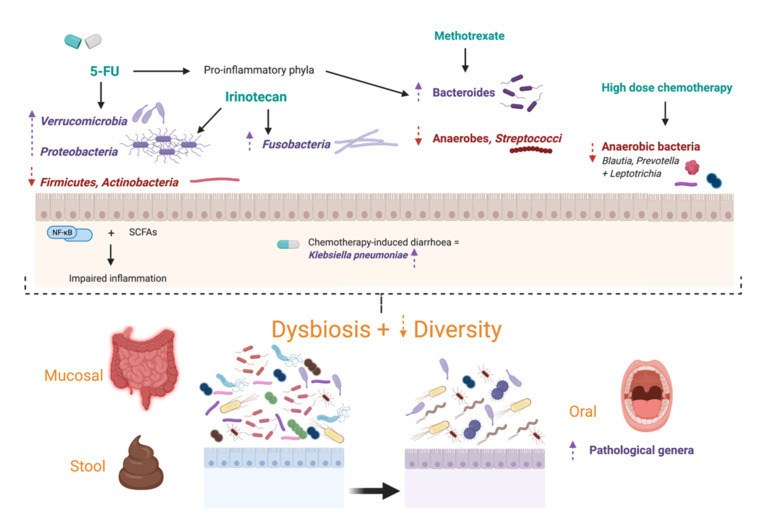
The impact of chemotherapy and immunotherapy on the microbiome. Dysbiosis with a reduction in diversity and operational taxonomic units is evidenced following different treatment regimes. This dysbiosis occur in colon mucosa, stool and oral cavity of the treated patients. The oral dysbiosis includes the enrichment of pathological genera such as *Neisseria* spp. The gut mucosal and stool dysbiosis include a specific shift towards pro-inflammatory bacteria with a reduction of anaerobic bacteria. Dotted arrows represent the shift in microbiome and bold arrows represent the effect of chemotherapy on the microbiome composition.

Despite the improved efficacy and survival with modern treatments, both the adverse effects and sequelae of chemotherapy represent a major cause for concern amongst patients and clinicians [51]. A salient concept to consider is that pathological disease states or, conversely, medical therapies may promote dysbiosis, thereby influencing clinical outcomes [Figure 2] [27].

Chemotherapy causes a disturbance in microbial community structure and is associated with a reduction in microbiome diversity as well as a decrease in the richness and abundance of operational taxonomic units [76,77]. A pre-clinical study revealed an enrichment of predominantly Gram-negative bacteria following administration of 5-FU in rats [78]. This was validated in more recent clinical studies showing an enrichment, in faecal samples, of Proteobacteria, reduction of Firmicutes, Actinobacteria and taxa that impair inflammation through modulating the NF-kB pathway and producing short-chain fatty acids [77,79]. Similarly, Galloway-Peña et al. [80] unveiled statistically significant increases in *Lactobacillus* with significant decreases in primarily anaerobic genera including *Blautia, Prevotella,* and *Leptotrichia* in buccal and faecal samples. A decrease in anaerobic bacteria was induced in both adult and paediatric patients undergoing conditioning and high-dose chemotherapy regimens, respectively [77,81].

### 3.1. Specific Chemotherapy Regimens

Various human and animal studies have illustrated that certain chemotherapy regimens result in dysbiosis. A recent study demonstrated a similar shift away from beneficial bacteria such as Actinobacteria and towards phyla with pro-inflammatory traits such as *Bacteroidetes* and *Verrucomicrobia* upon the administration of 5-FU in mice [52]. Such results are also seen with the administration of methotrexate in a rat model, which induced an absolute and relative decrease in anaerobes (13-fold) and *Streptococci* (296-fold) as well as a relative increase in *Bacteroides* [77]. Importantly, these changes were most prominent at the peak of mucositis severity clinically, and reduced bacterial presence was related to the presence of diarrhoea. Enrichment of inflammatory bacteria such as *Bacteroides* following chemotherapy has been shown in human faecal samples as well [81,82]. Chemotherapy-induced diarrhoea was recently found to be significantly associated with *Klebsiella pneumoniae* enrichment in patients with resected stage III CRC undergoing the CapeOX regimen [83]. A recent study demonstrated statistically significant differences in gut microbial abundance before and after chemotherapy with both XELOX and FOLFIRI regimens-including differences in the abundance of *Peptostreoptococcus, Clostridiales*, and *Prevotella* as well as altered gut fungi [21].

Various studies have reported statistically significant alterations in microbial composition following oxaliplatin administration. A study of 40 male BALB/c mice demonstrated increased abundance of Gram-negative bacteria in the gut following oxaliplatin administration, specifically a reduction in *Parabacteroides* and *Prevotella_1_* species and an increase in *Prevotella_2_* and *Odoribacter* in tissue samples [84]. These genera are from the *Bacteroides* phylum, which stems from the *Bacteroidetes* family—commensals to the GI tract but also opportunistic pathogens when the intestinal barrier is disrupted. Another tissue analysis of 40 Kunming female mice similarly demonstrated an increased abundance of *Bacteroidetes* and reduced abundance of *Prevotella* following oxaliplatin [85]. Interestingly, faecal samples from both mice and humans after taking probiotics had a lower abundance of *Bacteroides* and a higher abundance of *Prevotella* [85].

Furthermore, a pre-clinical study in Sprague-Dawley rats showed that irinotecan had a greater impact than 5-FU and oxaliplatin on the composition of faecal microbiota, but both chemotherapeutic drugs induced microbial and metabolic changes, activating inflammatory processes [86]. In particular, irinotecan was associated with an increased relative abundance of *Fusobacteria* and *Proteobacteria*. The latter has also been enriched in human studies [Table 1] [76,82]. There does not appear to be a specific bacterial community that is consistently altered following chemotherapy, most likely due to a lack of homogeneity of patient groups and sample types analysed, as well as a lack of standardization in laboratory protocols and computation methods. Nevertheless, various clinical and pre-clinical studies have demonstrated a shift away from the normal microbiota, emphasising that despite the heterogeneity in microbial shifts, chemotherapy does influence the gut microenvironment to cause dysbiosis. Future research with standardized protocols should be pursued to qualify and quantify this change accurately with a view to harnessing it to improve treatment outcomes and reduce adverse effects (Table 1 and Table 2).

### 3.2. Oral Dysbiosis

The alterations of the oral microbiota in the context of chemotherapy are not well established. A systematic review by Napeñas et al. [89] and prospective study by de Mendonca et al. [90] demonstrated shifts in the oral microbiota towards pathological genera such as *Streptococcus viridans*, Neisseria spp. and Candida spp. during cancer therapy. These findings support the hypothesis of oral dysbiosis in the setting of chemotherapy [55]. Alterations in the oral bacteriome were detected in mucosal samples but of greater magnitude in salivary communities, and though they correlated with mucositis severity, they were universal in the mouth—constant in healthy, erythematous and ulcerated sites. These studies also highlighted the paucity of current, longitudinal, well-controlled studies using highly sensitive high-throughput sequencing to characterise the oral micro-environment throughout chemotherapy. Disruption of the indigenous microbial community with growth of pathobionts and reduction of beneficial commensals would impair the ability of the mucosa to remain intact during an antineoplastic challenge [55]. The plausibility of this hypothesis was suggested by Perales-Puchalt et al. [91] in a murine model of intestinal mucositis in the context of the antineoplastic agent cisplatin. 16S rRNA sequencing analysis of faecal DNA confirmed that cisplatin induced measurable dysbiosis. This dysbiosis was characterised by significant increases in bacteria of the *Bacteroidaceae* and *Erysipelotrichaceae* families, as well as in *Bacteroides uniformis*. In contrast, cisplatin caused a significant decrease in *Ruminococcus gnavus*, a trans-sialidase-expressing bacterial strain that acquires nutritional competitive advantage by degrading mucins. Furthermore, gavage of faecal pellets overturned cisplatin-induced increases in *Bacteroidaceae* and *Erysipelotrichaceae* family bacteria.

In a similar way, these studies highlight chemotherapy-induced oral microbial dysbiosis but are unable to clarify the significance of these shifts on treatment efficacy. Further research that seeks to address these questions is needed to optimize patient outcomes.

### 3.3. Effects of Immunotherapy on the Microbiome

ICIs, specifically ipilimumab (anti-CTLA-4), tremelimumab (anti-CTLA-4) and nivolumab (anti-PD-1), have revolutionised cancer therapy [73]. Immunotherapy in CRC shows promise in improving patient outcomes, in particular nivolumab and pembrolizumab with metastatic dmmR-mSI-H CRC; however, the evidence to support its widespread use remains preliminary at this stage [10]. Nevertheless, it is prudent to characterise the effect immunotherapy has on the gut microbiome so that patient outcomes can be optimised when these agents become more commonly used.

Pre-clinical studies have demonstrated that immunotherapies cause a shift in the microbiota composition. The ground-breaking study by Vetizou et al. [68] analysing stool samples in recolonised GF and antibiotic-treated mice demonstrated that the microbiome composition following ipilimumab administration had enrichment of *Clostridiales* and reduced abundance of *Bacteroidales* and *Burkholderiales*. Furthermore, whilst the *Bacteroides* species was decreased in faeces, *Bacteroides thetaiotaomicron* and *Bacteroides uniformis* were enriched in mucosal samples from the small intestine, suggesting that the microbiome shifts following ICI treatment vary depending on samples studied. Analysis of stool samples following nivolumab treatment in patients with non-small-cell lung cancer (NSCLC) demonstrated enrichment of *Rikenellaceae, Prevotella, Streptococcus, Lactobacillus, Bacteroides plebeius, Oscillospira* and *Enterobacteriaceae* compared to healthy controls [92]. Another study of 11 NSCLC patients demonstrated a positive correlation between increased *Granulicatella* abundance and improved treatment response to nivolumab [93]. It also demonstrated higher abundance of commensals such as *Akkermansia muciniphila*, *Rikenellaceae*, *Bacteroides*, *Peptostreptococcaceae, Mogibacteriaceae* and *Clostridiaceae* in the controls than those in the patients receiving nivolumab. However, future studies with greater sample sizes are required to reproduce statistically significant results.

## 4. Effects of Synbiotics

Probiotics are defined by the World Health Organisation as “live microorganisms which when administered in adequate amounts confer a health benefit on the host” [94]. As a novel approach to augment cancer therapy, they are thought to improve the diversity profile of the intestinal microbiota and reduce the extent of chronic inflammation and production of carcinogenic material in dysbiosis [2,95,96]. Prebiotics are non-digestible food constituents that selectively alter the growth of certain host-beneficial bacteria [97]. The combination of probiotics and prebiotics is called synbiotics. Both prebiotics and probiotics have demonstrated ability to alter the commensal microbiota toward a beneficial composition and perhaps be used advantageously in patients with CRC [98]. Additionally, emerging evidence has shown their effects on the efficacy and toxicity of chemotherapy and immunotherapy.

### 4.1. Probiotics

Despite promising results being reported in some pre-clinical models, overall results for probiotics are largely inconsistent. For example, *S. thermophiles* TH-4 leads to attenuation of intestinal damage in non-tumour bearing rats treated with methotrexate and 5-FU [99,100]. However, a subsequent study in tumour-bearing rats treated with methotrexate was unable to demonstrate any beneficial effect [101]. Similarly, probiotic factors derived from *E. coli* Nissle 1917 and *Lactobacillus fermentum (L. fermentum)* BR11 partially protected the intestine from 5-FU-induced mucositis, and treatment with *Lactobacillus acidophilus (L. acidophilus)* improved the inflammatory and functional aspects of 5-FU-induced intestinal mucositis [102,103]. Additionally, oral probiotic *Lactobacillus rhamnosus (L. rhamnosus)* and *Bifidobacterium infantis (B. infantis)* prevented FOLFOX treatment-induced intestinal mucositis in a CRC-bearing mouse model and CRC-bearing rat model, respectively, thereby avoiding dose reduction caused by intestinal toxicity [2,104,105]. *L. rhamnosus* may also confer advantages in augmenting the anti-tumour response produced by immunotherapy. Owens et al. [106] showed that administration of *L. rhamnosus* GG (LGG) decreased tumour burden via increasing the CD8 T-cell response in a murine CRC model consistent with literature regarding immunotherapy for melanoma, suggesting that an absence of lactobacilli correlates with a poorer response to immunotherapy [107].

In clinical studies, the use of Yakult (*Bifidobacterium breve* strain, 10^9^ living bacteria) in children undergoing chemotherapy resulted in reduction in incidence of fever and prevented some modifications in gut microbiota such as an increase in *Enterobacteriaceae* [108]. In a single blinded RCT, Ishikawa et al. [109] investigated the effect of whether the probiotic *Lactibacillus casei* (*L. casei*) had any role in preventing tumour recurrence in a Japanese cohort of patients free from CRC having had 2 or more tumours removed in the past. They showed that *L. casei* reduced the atypia of tumours that recurred, resulting in reduced severity, but were unable to demonstrate a reduction in total new colorectal tumours developing. A larger study, which enlisted adults undergoing 5-FU regimens with *L. rhamnosus* supplementation, found a decreased incidence of Grade 3 or 4 diarrhoea and the need for chemotherapy dose reduction [110].

Promisingly, Benito et al. [111] demonstrated that microencapsulated probiotics *Bifidobacterium bifidum* (*B. bifidum*) and *Lactobacillus gasseri* (*L. gasseri*) reduced intestinal lesions and faecal occult blood loss in murine colorectal cancer (APCMin/+ mice), likely through inhibiting the Wnt/β-catenin signalling pathway. Furthermore, an even greater protective effect was conferred with the co-administration of the probiotics with the flavonoid quercetin [111]. *Bifidobacterium breve* (*B. breve*) is another probiotic of the *Bifidobacterium* spp. that has been shown to confer anti-tumour properties. Yoon et al.’s murine model isolated two specific *B. breve* species that were able to improve anti-tumour immunity when used in combination with anti-cancer therapeutics such as oxaliplatin and PD-1 blockade as measured through increased CD4+/Treg, CD8+/Treg and effector CD8+/Treg as well as increased intra-tumour cytokine expression [112]. Whilst these two trials show encouraging results, they need to be further tested in clinical trials to adequately assess the efficacy of probiotic use in augmenting the response to chemo- and immunotherapeutics in patient populations.

It is important to note that despite the generally accepted notion of probiotics being a safe food adjunct, with many probiotic products being granted a ‘generally regarded as safe’ status, probiotic bacteria can translocate from the gastrointestinal tract and result in clinically significant disease [113]. In healthy subjects, probiotic bacteria generally do not result in severe disease even when they do translocate. However, studies looking at immunocompromised populations suggest otherwise, with some reports of septic complications due to probiotics [113]. Additionally, there are a number of other challenges when conducting probiotic associated research. Most notably, humans exhibit individual, region and strain specific mucosal colonisation patterns resulting in an individualised impact on microbiota in response to probiotic administration [114]. Moreover, stool sample microbiome analysis only partially correlates with human gut mucosal microbiome, making predicting the individual response to probiotics especially challenging [114]. This notion challenges the application of a universal probiotic that can be applied to all patients with CRC and rather advocates for the research and development of personalised probiotic approaches to achieve optimal patient outcomes [114].

### 4.2. Prebiotics

Administration of certain prebiotics demonstrates beneficial shifts in the microbiome. For example, fructo-oligosaccharide administration has been shown to increase *Bifidobacteria* spp. in both rat studies and human studies [115,116]. In addition, fructo-oligosaccharide is also linked to increased mucin production [116]. Butyrate, as discussed previously, has been identified as a potential antineoplastic agent in the colon [117]. It plays an essential role in mucosal regeneration and in the inhibition of pro-inflammatory cytokine production [118,119]. Glutamine, which is known to be an effective gut protectant during stressful conditions, reduces the incidence and severity of late-onset diarrhoea following CPT-11 treatment in rats [120,121]. Water-soluble polysaccharide extracted from the sporoderm-removed spores of *Ganoderma Lucidum* (GLP) was shown to reduce inflammation-induced tumorigenesis and microbiota dysbiosis in an azoxymethane/dextran sulfate sodium (AOM/DSS) mouse model through a variety of mechanisms including modulation of endotoxaemia induced by the TLR4/MyD88/NF-kB pathway, strengthening colonic epithelial integrity and goblet cell function and increasing short-chain fatty acid production amongst others [122]. Guo et al. [122] therefore postulate that GLP might be an effective prebiotic treatment to use to ameliorate AOM/DSS induced tumorigenesis in CRC.

When looking specifically at improving chemotherapy outcomes, however, prebiotics have thus far not shown benefit. In an experimental study of mucositis induced by 5-FU in rats, *L. fermentum* reduced jejunal inflammation with no additional benefit added by the prebiotic fructo-oligosaccharide [123]. Furthermore, dietary fibre intervention has been shown to alter the composition of GI microbiota, specifically increasing the number of *Bifidobacterium* and *Lactobacillus* spp. as well as increasing faecal butyrate concentration in humans, thought to be beneficial in increasing the suppressing neoplastic activity of CRC [124]. However, despite prebiotic fibre intervention having a tumour-suppressive effect in a CRC gnotobiotic mouse model, clinical trials have not been able to demonstrate similar tumour-suppressive activity with dietary fibre intervention [125,126,127,128].

Synbiotics have been shown to be able to augment the microbiome; however, the clinical utility of this in CRC treatment is yet to be established. It is possible that prebiotics and probiotics will become a novel adjunct to anti-cancer treatment in the future; however, for now, it is prudent for further research to be conducted, especially in human studies, to qualify and quantify their treatment benefit.

## 5. Conclusions

Substantial evidence shows that the gut microbiota influences the efficacy of chemotherapy and severity of toxicity and facilitates chemotherapy resistance. Several studies have demonstrated a direct relationship between an intact microbiome, immune functioning and chemotherapy efficacy. Contrastingly, others have suggested that certain microbes, such as *Fn*, may increase chemoresistance to 5-FU and that dysbiosis has a negative correlation with chemotherapeutic outcomes. Additionally, the TIMER framework that was previously proposed by Alexander et al. [27] can been used to understand pharmacomicrobiomics in the context of CRC. On the other hand, chemotherapy has been reported to induce a dysbiosis in both humans and animals, with microbial shifts extending beyond colonic mucosa and involving the oral microenvironment. This is characterised by a shift away from eubiosis and towards inflammatory phyla such as *Bacteroidetes*. Ultimately, whilst there is growing evidence in mouse models, there is a major discrepancy and lack of evidence within clinical trials that support the use of prebiotics and probiotics to improve chemotherapy and immunotherapy outcomes.

Though findings in current literature are promising, a greater understanding of the exact relationship between the gut microbiota, host response and outcomes of anti-cancer treatment is warranted to ensure an individualised, more effective approach in the treatment of CRC and a reduction of associated toxicities.

## Figures and Tables

**Table 1:** Stool and Mucosal Dysbiosis following Chemotherapy in Humans.

Author	Study Subjects (n)	Specimen Types	Method	Microbiota Changes
Montassier, Gastinne, Vangay, Al-Ghalith, Bruley des Varannes and Massart [76]	Non-Hodgkin’s Lymphoma (28)	Stool	16S rRNA	↓ Firmicutes ↓ Actinobacteria ↑ Proteobacteria
Galloway-Peña, Smith, Sahasrabhojane, Ajami, Wadsworth, Daver, Chemaly, Marsh, Ghantoji, Pemmaraju, Garcia-Manero, Rezvani, Alousi, Wargo, Shpall, Futreal, Guindani, Petrosino, Kontoyiannis and Shelburne [80]	Acute myelogenous leukaemia patients (34)	Buccal specimens Stool	16S rRNA (V4)	↓ Oral and stool α-diversity (with carbapenem) ↓ *Lactobacillus* (oral and buccal) ↑ Anaerobes e.g., *Blautia, Prevotella, Leptotrichia*
Zwielehner, Lassl, Hippe, Pointner, Switzeny, Remely, Kitzweger, Ruckser and Haslberger [81]	Chemotherapy patients (17)	Stool	16S rRNA	↓ Diversity of *Clostridium* clusters *IV* and *XIVa* ↑ *Bacteroides* ↓ Bifidobacterium
Fei, Lijuan, Xi, Wei, Jing, Miao and Shuwen [83]	Resected stage III CRC patients undergoing CapeOX regimen (17)	Stool	16S rRNA	↓ Microbial richness and diversity ↑ *Klebsiella pneumoniae*
Vanlancker, Vanhoecke, Stringer and Van de Wiele [82]	In vitro mucosal stimulator treating stool and mucosa with 5-FU and SN-38 (active metabolite of irinotecan) (6 human donors)	Stool Mucosal	16S rRNA (V3–4)	↑ Proteobacteria (*Escherichia/Shigella)* ↑ Bacteroidetes (*Bacteroides*) ↑ Firmicutes (*Clostridium* cluster *XIVa, Veillonella*) ↑ *Bacteroides* ↓ *Escherichia/Shigella*

Abbreviations: ↑, increased; ↓, decreased.

**Table 2:** Stool and Mucosal Dysbiosis following Chemotherapy in Animals.

Author	Study Subjects (n)	Specimen Types	Method	Microbiota Changes
Fijlstra et al. [77]	Rats treated with methotrexate	Stool	FISH	↓ Anaerobes ↓ Streptococci ↑ *Bacteroides*
Forsgard, Marrachelli, Korpela, Frias, Collado, Korpela, Monleon, Spillmann and Osterlund [86]	Sprague-Dawley rats injected with 5-FU, oxaliplatin or irinotecan (48)	Stool	16S rRNA	Irinotecan: ↑ Fusobacteria ↑ Proteobacteria 5-FU and Oxaliplatin caused minor shifts
Stringer et al. [87]	Irinotecan-treated rats (81)	Stool	DNA extraction + PCR	↑ *E. coli* ↑ *Staphylococcus* spp. ↑ *Clostridium* spp. ↓ *Lactobacillus* spp. ↓ *Bifidobacterium* spp. ↓ *Bacteroides* spp.
Lin et al. [88]	Tumour-bearing rats receiving irinotecan +/− oral glutamine bolus (6) Rats receiving two cycles of irinotecan followed by 5-FU (6)	Stool	DNA extraction + PCR	↑ *Clostridium* cluster *XI* ↑ *Enterobacteriaceae* Glutamate caused: ↓ *Clostridium cluster VI* ↓ *Bacteroides*
Carvalho, Vaz, Pereira, Dorella, Aguiar, Chatel, Bermudez, Langella, Fernandes, Figueiredo, Goes-Neto and Azevedo [52]	5-FU treated mice (72)	Stool	16s rRNA (V4)	↑ *Bacteroidetes* ↑ *Firmicutes* ↑ *Proteobacteria*

Abbreviations: ↑, increased; ↓, decreased.
